# QM/MM Calculations with deMon2k

**DOI:** 10.3390/molecules20034780

**Published:** 2015-03-16

**Authors:** Dennis R. Salahub, Sergei Yu. Noskov, Bogdan Lev, Rui Zhang, Van Ngo, Annick Goursot, Patrizia Calaminici, Andreas M. Köster, Aurelio Alvarez-Ibarra, Daniel Mejía-Rodríguez, Jan Řezáč, Fabien Cailliez, Aurélien de la Lande

**Affiliations:** 1 Department of Chemistry, CMS—Centre for Molecular Simulation, IQST—Institute for Quantum Science and Technology and ISEEE—Institute for Sustainable Energy, Environment and Economy, University of Calgary, 2500 University Drive NW, Calgary, AB T2N 1N4, Canada; E-Mail: zhangrui1002@gmail.com; 2 Department of Biological Science and CMS—Centre for Molecular Simulation, University of Calgary, 2500 University Drive NW, Calgary, AB T2N 1N4, Canada; E-Mails: snoskov@ucalgary.ca (S.Y.N.); nvan.ucalgary@gmail.com (V.N.); 3 School of Applied Sciences and Health Innovation Research Institute, RMIT University, Melbourne, VIC 3001, Australia; E-Mail: lev.bogdan@gmail.com; 4 Institut Charles Gerhardt, UMR 5253 CNRS/UM2/ENSCM/UM1, 8 rue de l’Ecole Normale, Montpellier 34296, France; E-Mail: annick.goursot@gmail.com; 5 Departamento de Química, CINVESTAV, Centro de Investigación y de Estudios Avanzados, Av. Instituto Politécnico Nacional, 2508, A.P. 14-740, México D.F. 07000, Mexico; E-Mails: pcalamin@cinvestav.mx (P.C.); akoster@cinvestav.mx (A.M.K.); aalvarezi@cinvestav.mx (A.A.-I.); dmejia@cinvestav.mx (D.M.-R.); 6 Institute of Organic Chemistry and Biochemistry and Gilead Science and IOCB Research Center, Academy of Sciences of the Czech Republic, Flemingovo nam. 2, Prague 6 166 10, Czech Republic; E-Mail: rezac@uochb.cas.cz; 7 Laboratoire de Chimie Physique—CNRS UMR 8000, Université Paris-Sud, Bật. 349, Campus d’Orsay. 15, rue Jean Perrin, Orsay Cedex 91405, France; E-Mails: fabien.cailliez@u-psud.fr (F.C.); aurelien.de-la-lande@u-psud.fr (A.L.)

**Keywords:** Quantum Mechanical/Molecular Mechanical, QM/MM, density functional theory, deMon2k software, biomolecular modeling, auxiliary DFT, double asymptotic expansion, constrained DFT

## Abstract

The density functional code deMon2k employs a fitted density throughout (Auxiliary Density Functional Theory), which offers a great speed advantage without sacrificing necessary accuracy. Powerful Quantum Mechanical/Molecular Mechanical (QM/MM) approaches are reviewed. Following an overview of the basic features of deMon2k that make it efficient while retaining accuracy, three QM/MM implementations are compared and contrasted. In the first, deMon2k is interfaced with the CHARMM MM code (CHARMM-deMon2k); in the second MM is coded directly within the deMon2k software; and in the third the Chemistry in Ruby (Cuby) wrapper is used to drive the calculations. Cuby is also used in the context of constrained-DFT/MM calculations. Each of these implementations is described briefly; pros and cons are discussed and a few recent applications are described briefly. Applications include solvated ions and biomolecules, polyglutamine peptides important in polyQ neurodegenerative diseases, copper monooxygenases and ultra-rapid electron transfer in cryptochromes.

## 1. Introduction—Why deMon2k?

Since the pioneering work of Warshel and Karplus [1] and Warshel and Levitt [2] in the seventies many implementations of hybrid Quantum Mechanical/Molecular Mechanical (QM/MM) methodologies have ensued (see refs. [3,4,5,6] for recent reviews). This mini-review focuses exclusively on our recent work using the deMon2k Density Functional software [7] as the QM engine. deMon stands for density of Montreal since the earliest versions of the code were developed in the Salahub group at the Université de Montréal, in the late 1980s. A brief description of the program and its history may be found on the demon-software web site [7]. The current version of deMon2k is maintained and developed by a loose international consortium known as the deMon Developers with a principal node at CINVESTAV in Mexico City handling licensing (free for academics) and distribution (see the web site for details). The main features of the public release of deMon2k are:

- Variational fitting of the Coulomb potential
- Auxiliary density functional theory (ADFT)
- Adaptive numerical integration for exchange-correlation functionals
- Analytical molecular integral recurrence relations without limitation
- Half-numerical ECP and MCP integral recurrence relations
- MinMax self-consistent field (SCF) stabilization and acceleration
- Empirical dispersion corrections for all elements
- Geometry optimization with restricted step algorithm
- Hierarchical transition state finder
- Intrinsic reaction coordinate calculation
- Born-Oppenheimer molecular dynamics (BOMD) simulations
- Time-dependent ADFT (TD-ADFT)
- Auxiliary density perturbation theory (ADPT)
- Electric moments, polarizabilities and hyperpolarizabilities
- Nuclear magnetic resonance (NMR), infra-red (IR) and Raman spectra
- Thermodynamic data from polyatomic ideal gas model
- Population analyses (Mulliken, Löwdin, NBO, Bader)
- Topological analysis of molecular fields
- Interfaces for visualization software (Molden, Molekel, Vu)
- Portability to various computer platforms and operating systems
- Parallelized code (MPI)
- DFT optimized basis sets
- Automatic generation of adaptive auxiliary function sets

Additionally, the current developer version of deMon2k (4.2.5) adds the following features to the list:

- Molecular mechanics energies, gradients and Hessian
- QM/MM interface to CHARMM, Cuby and PUPIL
- Constrained DFT and ADFT energies and gradients
- Asymptotic molecular integral expansions for mixed SCF
- Exact exchange with three-center electron repulsion integrals (ERIs)
- X-ray absorption and emission spectroscopy
- ROKS perturbation theory and Fukui functions
- Magnetizability, rotational g-tensor and spin-spin coupling constants
- BOMD property (NMR, , ) and analysis tools
- Non-iterative CPKS solver for perturbation theory
- VMT, LB94, B3LYP, PBE0 and M06-2X functionals
- Hirshfeld (iterative), Becke and Voronoi population analyses
- Plotting of Fukui functions, induced magnetic fields and perturbed or deformed densities

Further details concerning theory, implementation and program features may be found in the deMon U ser’s guide [7]. The current deMon2k developer version can also be obtained from the CINVESTAV group (mail to: pcalamin@cinvestav.mx) or from a deMon2k developer.

A *Software Focus* on deMon2k appeared in the Wiley WIRES series in 2011 [8]. That paper focused on the main methodological and implementation underpinnings of deMon2k, starting from the pioneering work on density-fitting of Dunlap, Connolly and Sabin [9] and proceeding through the formulation of Auxiliary DFT [10], in which the fitted density is used for both the Coulomb terms and the exchange-correlation terms. This lies at the heart of the efficiency of deMon2k and has allowed its extension to large systems, now containing a thousand or more atoms and tens of thousands of basis functions, or for the use of deMon2k in Born-Oppenheimer simulations containing a hundred or more atoms for tens or hundreds of picoseconds. In deMon2k the auxiliary, fitting, basis set consists of Hermite Gaussians (advantage is taken of the Hermite recurrence relationship) and it may be determined automatically from the orbital basis set (the most recent orbital basis sets have been optimized at the GGA (Generalized Gradient Approximation) level) [11]. Efficient calculation of the electron repulsion integrals (ERIs) within the ADFT formalism represents a major accomplishment of the CINVESTAV group; a landmark being the specially optimized recurrence relations for three-center ERIs that take advantage of the auxiliary function sets and their primitive Hermite Gaussian form [12]. The exchange-correlation terms require numerical integration on a grid and for this deMon2k offers the option of adaptive grids that are generated to give the best accuracy for a given grid size [13]. Because the variational fitting of the Coulomb potential converges from below it is necessary to use a MinMax iterative scheme [14] in the SCF cycles (earlier codes that ignored this aspect often had convergence difficulties). With modern computer architectures, parallelization is imperative; deMon2k uses the Message Passing Interface in such a way that the efficiency of the serial code is preserved [15]. Some early benchmarks were given in the WIRES paper; others appear below.

The above-described features of deMon2k represent the state of play in 2011. Since that time, several key advances have been made that are crucial for the use of deMon2k for calculations and simulations on very large systems. These more recent features are described in somewhat greater detail in Section 2. They include the double asymptotic expansion for three-center ERIs [16], the asymptotic expansion for electrostatic embedding integrals [17] and, most recently a mixed SCF procedure that uses either direct or standard (store and retrieve) SCF approaches depending on whether the ERIs are of the near-field or far-field type [18].

In Section 3, Section 4 and Section 5, three different QM/MM implementations are compared and contrasted as to their strengths, weaknesses and areas of applicability: (i) an interface between deMon2k and the CHARMM molecular mechanical software; (ii) a version coded directly in deMon2k and (iii) a multipurpose framework for composite calculations called Cuby (Chemistry in Ruby). For each of these the main features of the methodology and its implementation are presented, pointing out pros and cons and setting the bounds on system size *etc.* for its intended use. This is complemented for each by brief descriptions of one or two representative applications from the recent literature.

## 2. Recent Developments of deMon2k that Permit QM/MM Simulations of very Large Systems

Most QM/MM calculations consist of QM regions with a few dozen to a few hundred QM atoms and MM regions with some thousands to a few million MM atoms. In this setup the MM atoms usually give rise to an electrostatic embedding which, in the simplest case, is a point charge embedding of the QM region. The corresponding energy expression is given by:

(1) $$E=E^{\left(0\right)}-\overset{QM}{\underset{a,b}{\sum }}\overset{MM}{\underset{D}{\sum }}P_{ab}\;\langle ab|{\hat{A}}_{D}\left(0\right)\rangle \;Q_{D}+\;\overset{QM}{\underset{A}{\sum }}\overset{MM}{\underset{D}{\sum }}\frac{Z_{A}Q_{D}}{\left|\vec{A}-\vec{D}\right|}$$

Similarly we find for an element of the modified core Hamiltonian:

(2) $$H_{ab}=H_{ab}^{\left(0\right)}-\overset{MM}{\underset{D}{\sum }}\;ab|{\hat{A}}_{D}\left(0\right)\;Q_{D}$$

In our implementations $E^{\left(0\right)}$ and $H_{ab}^{\left(0\right)}\;$ are usually the ADFT energy and core Hamiltonian matrix elements of the QM region, respectively [19]. The atomic orbitals a and b are (contracted) Cartesian Gaussian type orbitals (GTOs) in deMon2k and $P_{ab}$ denotes the corresponding density matrix element. The vectors $\vec{A}$ and $\vec{D}$ give the positions of the QM atom A with nuclear charge $Z_{A}$ and of the MM atom D with charge $Q_{D}$, respectively. The nuclear attraction type operator ${\hat{A}}_{D}$ is defined as:

(3) $${\hat{A}}_{D}\left(k\right)={\left(\frac{\partial }{\partial D_{x}}\right)}^{k_{x}}{\left(\frac{\partial }{\partial D_{y}}\right)}^{k_{y}}{\left(\frac{\partial }{\partial D_{z}}\right)}^{k_{z}}\frac{1}{\left|\vec{r}-\vec{D}\right|}$$

This general definition permits immediately the inclusion of MM atoms with higher point moments, e.g., dipole or quadrupole moments, in the embedding. For clarity of presentation we restrict ourselves here to point charges, *i.e.*, $=\left(k_{x},\;k_{y},k_{z}\right)=0$. Thus, ${\hat{A}}_{D}\left(0\right)$ denotes $1/\left|\vec{r}-\vec{D}\right|$. Note the sum over D in the second term of Equations (1) and (2) which runs over all MM atoms. Even though these terms have to be calculated only once in the self-consistent field (SCF) procedure, it can become a computational bottleneck for a very large number of MM atoms if nuclear attraction integral (NAI) recurrence relations are used [16]. Due to the very slow decay of Coulomb interactions an efficient screening of these integrals is not possible without jeopardizing the accuracy of the methodology. However, it is possible to separate the NAIs in the second term of Equations (1) and (2) into near-field and far-field integrals depending on the distances of the MM atoms from the QM atoms. To do so the extensions of the atomic orbitals involved in the NAI are calculated according to a user-defined threshold for the molecular integral accuracy (default value is 10^−10^ a.u.) [12]. These extensions are compared to the distances between the orbital centers (QM atoms) and the MM atoms. If the MM atom is located inside one of these extensions, the NAI is classified as near-field. Otherwise, it is a far-field NAI.

### 2.1. Asymptotic Expansion of QM/MM Embedding Integrals

For typical QM/MM calculations with large MM regions, the number of near-field NAIs that must be calculated by integral recurrence relations [20] is much smaller than the number of far-field NAIs. These far-field NAIs can be expressed to any desired accuracy by asymptotic integral expansions [17] of the form:

(4) $$\langle ab|{\hat{A}}_{D}{\left(0\right)\rangle }^{A}\;\sim \;\underset{m_{x}}{\sum }\underset{m_{y}}{\sum }\underset{m_{z}}{\sum }\frac{{\left(-1\right)}^{m}}{m_{x}!m_{y}!m_{z}!}{\left(\frac{\partial }{\partial D_{x}}\right)}^{m_{x}}{\left(\frac{\partial }{\partial D_{y}}\right)}^{m_{y}}{\left(\frac{\partial }{\partial D_{z}}\right)}^{m_{z}}\frac{1}{\left|\vec{A}-\vec{D}\right|}\;\langle a+m|b\rangle$$

The superscript A on the left-hand side of Equation (4) indicates the center of the asymptotic NAI expansion (an equivalent expansion exists for center B, too). The order of the asymptotic expansion is given by $m=\;m_{x}+\;m_{y}+m_{z}$. In deMon2k it is set to eight. The last term on the right-hand side of Equation (4), 〈a+m|b〉, represents an overlap integral in which the angular momentum index of orbital a is augmented by the asymptotic expansion order m, *i.e.*, $a+m=\left(a_{x}+m_{x},a_{y}+m_{y},a_{z}+m_{z}\right)$. Note that, except for the overlap integral, all terms of the asymptotic expansion relate to atoms rather than orbitals. Thus, there are two major differences of the asymptotic expansion with respect to regular NAI recurrence relations. First, nuclear attraction type integrals are turned into overlap type integrals. As a result, the calculation of the incomplete gamma function is completely avoided in the asymptotic expansion. Second, the information of the MM atom has been factored out of the molecular integral. Thus, the following asymptotic expansion for an element of the embedding core Hamiltonian, Equation (2), is possible:

(5) $$H_{ab}\sim \;H_{ab}^{\left(0\right)}-\overset{Near}{\underset{D}{\sum }}\;\langle ab|{\hat{A}}_{D}\left(0\right)\rangle \;Q_{D}-\;\underset{m_{x}}{\sum }\underset{m_{y}}{\sum }\underset{m_{z}}{\sum }\frac{{\left(-1\right)}^{m}}{m_{x}!m_{y}!m_{z}!}\;\langle a+m|b\rangle \;\overset{Far}{\underset{D}{\sum }}Q_{D}T_{AD}(m)$$

with:

(6) $$T_{AD}(m)={\left(\frac{\partial }{\partial D_{x}}\right)}^{m_{x}}{\left(\frac{\partial }{\partial D_{y}}\right)}^{m_{y}}{\left(\frac{\partial }{\partial D_{z}}\right)}^{m_{z}}\frac{1}{\left|\vec{A}-\vec{D}\right|}$$

**Figure 1:**
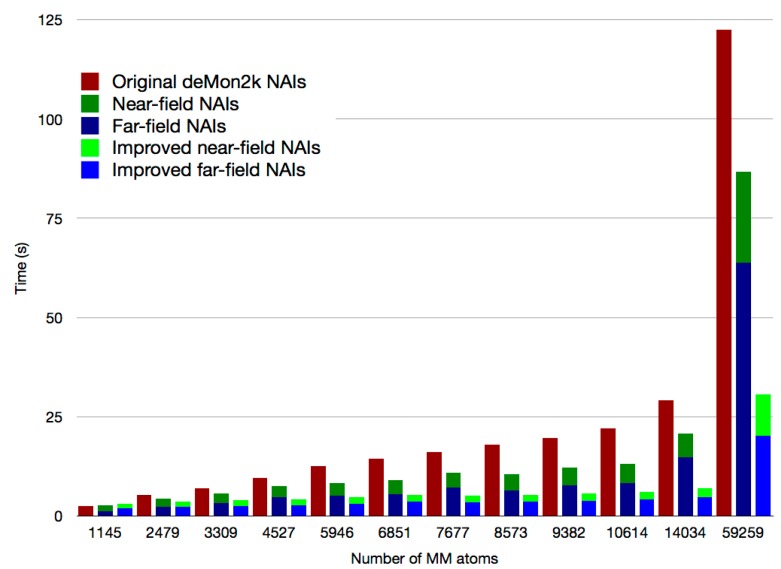
NAI calculation time for selected QM/MM systems using the original deMon2k code (red bars), the asymptotic expansion (dark blue and dark green bars) and the asymptotic expansion with the improved distance calculation algorithm (light blue and light green bars). Times refer to parallel runs with 12 cores.

Since $T_{AD}(m)$ depends only on atomic quantities, its calculation can be performed in the outer loops of the algorithm achieving an additional speedup. The separation into near- and far-field NAIs requires the recurrent comparison of atomic distances. Thus, an efficient algorithm must include a reduced calculation of such distances. A major improvement [21] has been recently achieved by using auxiliary fields for the definition of the MM atoms in the near- and far-field region of a given QM atom pair even before entering the orbital loop. With this auxiliary field, the atomic loops for the MM atoms, represented in the equations as the sum over *D*, are considerably reduced thus avoiding redundant distance calculations between the QM and MM atoms in the orbital loops, represented by the sums over a and b in Equation (1). Figure 1 depicts the calculation time of NAIs for the core Hamiltonian of a QM/MM system with 124 QM atoms and different numbers of MM atoms. The red bars show the calculation time of NAIs without considering the near- and far-field separation. The dark blue and dark green bars show the calculation time for the NAIs separated by near- and far-field regions with the original distance computation scheme [17]. Finally the light blue and light green bars show the near- and far-field NAIs calculation time with the new distance computation scheme. Using the asymptotic expansion of far-field NAIs already shows a reduction of the total computation time, being for the case of 14034 MM atoms almost one half. However, the improvement of the distance computation scheme further reduces the calculation time to a quarter of the standard NAI calculation and has a much better time growth behavior than the previous implementation. This underlines the importance of having in mind the difficulty of the distance calculation in the algorithm. More details of the implementation of the asymptotic NAI expansion are given in Ref. [17].

### 2.2. Double Asymptotic Expansion of Electron Repulsion Integrals

In QM/MM calculations not only the point charge embedding but also the QM system itself can become rather large. Currently deMon2k can perform calculations with QM systems up to a couple of thousand atoms. If such setups are used in ADFT QM/MM calculations, another bottleneck may arise from the calculations of the electronic repulsion integrals (ERIs) inside the QM region. This can be seen immediately from the ADFT SCF energy expression:

(7) $$E^{SCF}=\;\overset{QM}{\underset{a,b}{\sum }}P_{ab}H_{ab}+\overset{QM}{\underset{a,b}{\sum }}\overset{QM}{\underset{\bar{c}}{\sum }}P_{ab}\langle ab\|\bar{c}\rangle \;x_{\bar{c}}-\frac{1}{2}\;\overset{QM}{\underset{\bar{c},\bar{d}}{\sum }}x_{\bar{c}}x_{\bar{d}}\;\langle \bar{c}\|\bar{d}\rangle +\;E_{xc}[\tilde{\rho }]$$

Here $P_{ab}$ and $H_{ab}$ denote elements of the density and core Hamiltonian matrix, $x_{\bar{c}}$ and $x_{\bar{d}}$ Coulomb fitting coefficients from the variational density fit and $E_{xc}[\tilde{\rho }]$ the exchange-correlation functional calculated with the fitted density,

(8) $$\tilde{\rho }\left(\vec{r}\right)=\;\overset{QM}{\underset{\bar{c}}{\sum }}x_{\bar{c}}\;\bar{c}\left(\vec{r}\right).$$

The second and third term in Equation (7) describe the electronic Coulomb repulsion in the QM system as obtained from the variational density fitting. In the short hand ERI notation the symbol ∥ in $\langle ab\|\bar{c}\rangle$ and $\langle \bar{c}\|\bar{d}\rangle$ denotes the two-electron repulsion operator $1/\left|{\vec{r}}_{1}-{\vec{r}}_{2}\right|$. It also separates the functions from electron 1 on the left side of ∥ from those of electron 2 on the right side. The three-index ERIs introduce a formal cubic scaling into the energy expression that can become a computational bottleneck for large QM regions. As was the case for the NAIs, it is possible to separate these ERIs into near- and far-field integrals depending on the distance of the charge density centers in Equation (9). In this case, the asymptotic expansion of the electrostatic potential of the primitive auxiliary function is used to define the near-field potential extension [12]. For QM systems widespread in space the number of near-field ERIs that must be calculated is much smaller than the number of far-field ERIs, the latter being the potential bottleneck in the computation. Thus, in deMon2k a double asymptotic expansion has been implemented for the calculation of the far-field ERIs as follows [16]:

(9) $$\langle ab\|\bar{c}\rangle =\;\iint^{\text{​}}a\left({\vec{r}}_{1}\right)\;b\left({\vec{r}}_{1}\right)\frac{\bar{c}({\vec{r}}_{2})}{\left|{\vec{r}}_{1}-{\vec{r}}_{2}\right|}\;d{\vec{r}}_{1}d{\vec{r}}_{2}$$

(10) $${\langle ab\|\bar{c}\rangle }^{A}∼{\left(\frac{\pi }{\zeta_{\bar{c}}}\right)}^{\frac{3}{2}}\underset{m_{x}}{\sum }\underset{m_{y}}{\sum }\underset{m_{z}}{\sum }\frac{{\left(-1\right)}^{m}}{m_{x}!m_{y}!m_{z}!}\;T_{AC}\left(m+\bar{c}\right)\;\langle a+m|b\rangle$$

The notation is analogous to the asymptotic NAI expansion with $\zeta_{\bar{c}}$ being the exponent of the Hermite Gaussian auxiliary function $\bar{c}$. Again, the advantages of using the double asymptotic expansion of far-field ERIs are the reduction of the two-electron electrostatic interaction integrals to analytically solvable modified overlap integrals of the type 〈a+m|b〉 and the factorization of the information about the auxiliary function potential to the atomic level in the function $T_{AC}\left(m+\bar{c}\right)$.

**Figure 2:**
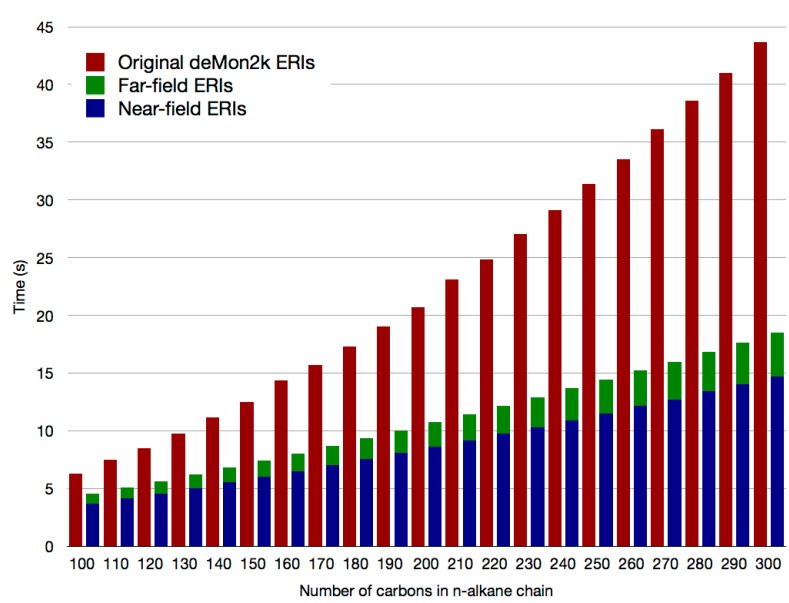
ERI calculation time per SCF cycle for selected n-alkane chains using the original deMon2k code (red bars) and the double asymptotic expansion (blue and green bars). Times refer to parallel runs with 12 cores.

As can be seen from Figure 2 the double asymptotic expansion reduces significantly the calculation time of the numerous far-field ERIs. As a result, the repetitive calculation of the relatively few near-field ERIs in the direct SCF (blue bars in Figure 2) can become the new (reduced) computational bottleneck in QM/MM calculations with large QM regions.

### 2.3. The Mixed SCF Scheme

To circumvent the near-field ERI computational bottleneck a new mixed SCF scheme has recently been implemented in deMon2k [18]. In the mixed SCF scheme the near-field ERIs are stored in memory whereas the far-field ERIs are calculated repeatedly using the efficient double asymptotic expansion. The main difference to other modified SCF schemes is that the determination of the integrals that will be stored in memory is based on their separation into near-field and far-field ERIs. To avoid any I/O bottleneck near-field ERIs will only be stored in RAM and not on a hard-disk.

The mixed SCF scheme is implemented in the parallel versions of deMon2k. When this scheme is applied, the program determines the necessary amount of RAM for the allocation of all the working fields, e.g., the density and Kohn-Sham matrices, the Coulomb vector and the work fields for the ERI calculation. After this, the amount of free RAM in the slave processors of the computational network for the calculation is determined, and this free RAM is then used for the near-field ERI storage.

**Figure 3:**
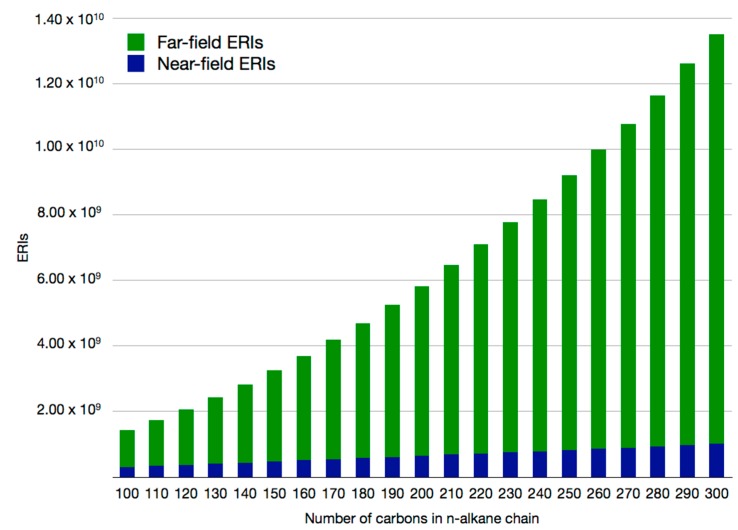
Number of near-field and far-field ERIs in selected *n*-alkane chains.

**Figure 4:**
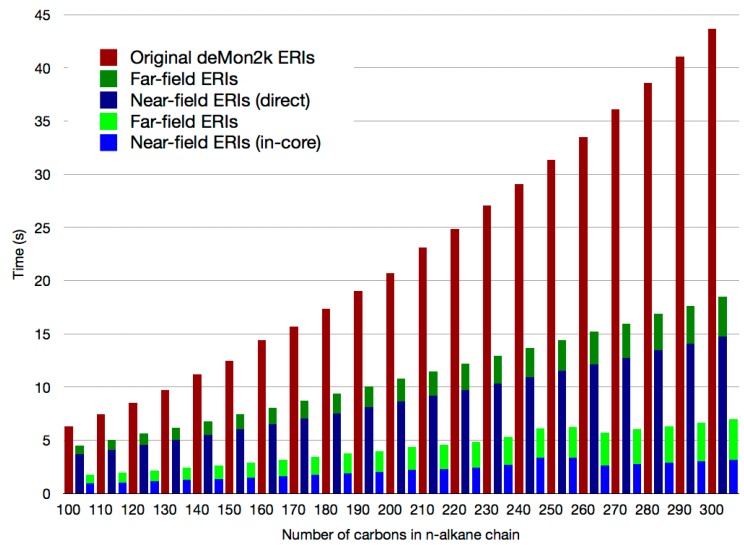
ERI calculation time per SCF cycle for selected *n*-alkane chains using the original deMon2k code (red bars), the direct SCF scheme with double asymptotic ERI expansion (dark blue and dark green bars) and the mixed SCF scheme (light blue and light green bars). Times refer to parallel runs with 12 cores.

In this way, the ERI calculation and storage is distributed over the network, achieving further efficiency for the computation and use of ERIs. In Figure 3 the number of near-field and far-field ERIs in the studied *n*-alkane chains is depicted. Since growth in the number of near-field ERIs is linear, increasing the number of atoms in the system will require only the addition of more processors to the computational network for the storage of the additional ERIs. This is possible since far-field ERIs do not contribute to the formal RAM requirement for allocation. Figure 4 shows benchmarks for the ERI calculation time in the n-alkane systems. Using the mixed SCF the near-field ERI bottleneck disappears. Thus, the ERI evaluation bottleneck in QM/MM calculations with large QM regions has been removed.

The implementation of the asymptotic expansion for the electrostatic embedding and the mixed SCF scheme for the ERI calculation gives deMon2k the capability of handling molecular systems for QM/MM calculations with very large MM regions (hundreds of thousands of atoms) and QM regions with several hundreds to a thousand atoms in parallel MPI calculations. This approach is also beneficial for Born-Oppenheimer molecular dynamics (BOMD) simulations of QM systems with up to around 200 atoms.

### 2.4. Exact Exchange in QM/MM Calculations

The election of hybrid functionals to describe the QM region in QM/MM calculations restricts considerably the size of this system due to the large computational cost of evaluating the exact exchange term. In order to address this problem a new formulation of variationally-fitted exact exchange has been recently implemented in deMon2k [22]. The new implementation is called Local Density-Fitting Exact Exchange (LDF-EXX) and it is similar in essence to the local correlation methods of Pulay and Saebo [23]. In LDF-EXX the exchange energy is given by:

(11) $$E^{LDF-EXX}=\;-\frac{1}{2}\overset{QM}{\underset{a,b}{\sum }}P_{ab}X_{ab}=-\frac{1}{2}\overset{QM}{\underset{a,b}{\sum }}\overset{QM}{\underset{\bar{c},\bar{d}}{\sum }}\overset{QM}{\underset{i}{\sum }}P_{ab}\langle ai\|\bar{c}\rangle \langle \bar{c}\|{\bar{d}\rangle }^{-1}\langle \bar{d}\|ib\rangle$$

Here the sum over i is for all occupied molecular orbitals of the QM region. The particularity of LDF-EXX is that the molecular orbitals are localized in space, allowing the restriction of atomic orbitals and auxiliary functions only to those centered nearby to i.

**Figure 5:**
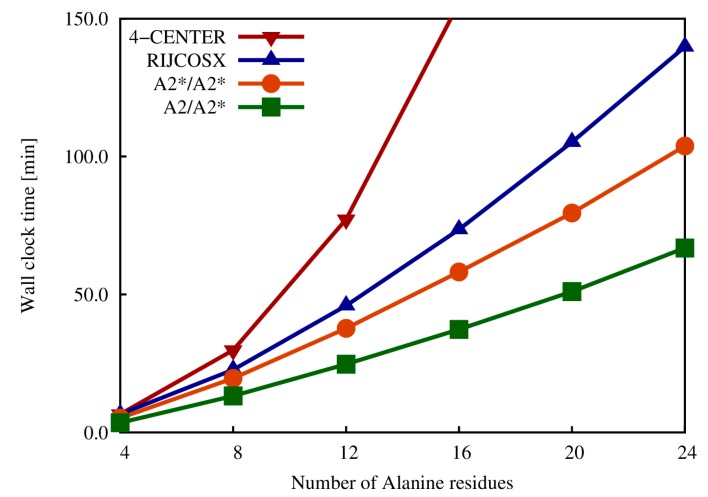
Total SCF times for a series of polyalanine right-handed alpha helices using a standard four-center ERI approach (red line), the resolution-of-the-identity with chains of spheres exchange method (blue line) and the LDF-EXX as implemented in deMon2k with different combinations of auxiliary function sets (orange and green lines).

The reduction of computational complexity achieved by LDF-EXX is depicted in Figure 5, where a comparison between the total SCF times for serial Hartree-Fock calculations of a series of polyalanine (polyA) right-handed alpha helices computed with different methodologies is shown. Here, total SCF time refers to the time to converge the wave function which occurred after 10 SCF cycles in all methodologies. In Figure 5, the two lines of LDF-EXX labeled as A2/A2* (green) and A2*/A2* (orange) show speed-ups of, at least, 20 times compared to a standard four-center ERI approach. Furthermore, comparison to the computationally efficient resolution-of-the-identity chains of spheres exchange (RIJCOSX) algorithm [24] shows that LDF-EXX has lower scaling behavior and is, at least, 30% faster.

The appearance of two auxiliary function sets in the LDF-EXX labels follows from the flexibility of the code to choose one auxiliary function set for the SCF iterations and another, generally larger, to calculate a non-self-consistent energy. In this way a better accuracy-performance relation is achieved. The A2/A2* LDF-EXX takes only 3 min per SCF iteration for the Ala_24_ alpha helix, which consists of almost 250 QM atoms. This result shows that hybrid DFT calculations of QM regions with several hundreds of atoms are feasible with the new LDF-EXX implemented in deMon2k.

## 3. CHARMM-deMon Interface

While there are many different QM/MM interfaces available to a number of molecular simulation softwares, compatibility with CHARMM [25] allows access to a broad range of simulation techniques including compatibility with the latest additive force-fields for proteins, nucleic acids, lipids and small molecules, polarizable force-fields such as the Drude polarizable force-field and links to free energy simulation techniques (Thermodynamic Integration (TI), Umbrella Sampling (US) and Free Energy Perturbation (FEP)). In addition to advantageous developments we can also rely on the enhanced sampling algorithms implemented in CHARMM versions c36 to c39, allowing efficient system propagation in a very complex energy space. For example, an FEP calculation for QM/MM systems with solvent molecules allows a direct evaluation of *relative* thermodynamic properties while avoiding the limitations of classical potential functions. Thus, FEP calculations based on QM/MM could directly aid force-field development, as well as having an impact on multiple areas of interest e.g., ion solvation and ion transport in nanopores.

The particular QM/MM scheme employed in the interface between CHARMM and deMon2k is based on the work of Field *et al.* [26] and Woodcock *et al.* [25] adopting an additive scheme. Following the same notations proposed by Field *et al.*, the effective Hamiltonian of the entire system is formalized as:

(12) $${\hat{H}}_{eff}=\;{\hat{H}}_{QM}+\;{\hat{H}}_{MM}+\;{\hat{H}}_{QM/MM}$$

where ${\hat{H}}_{QM}\;$ is the pure Hamiltonian of the QM subsystem including the link atom(s), ${\hat{H}}_{MM}$ is the pure classical Hamiltonian described by the force field, and ${\hat{H}}_{QM/MM}$ is the Hamiltonian accounting for the coupling between the two subsystems.

According to the electrical embedding formula, ${\hat{H}}_{QM/MM}$ is given as

(13) $${\hat{H}}_{QM/MM}=\;-\underset{i,M}{\sum }\frac{q_{M}}{r_{iM}}+\;\underset{A,M}{\sum }\frac{Z_{A}q_{M}}{R_{AM}}+\;\underset{A,M}{\sum }\left(\frac{A_{AM}}{R_{AM}^{12}}-\;\frac{B_{AM}}{R_{AM}^{6}}\right)$$

where the first term is a single-electron operator generated by the external MM point charges, the second term describes the Coulomb interaction between the QM nuclei and external MM charges and the last accounts for the Pauli repulsion and van der Waals attraction between QM and MM atoms in the Lennard-Jones formalism.

In the implementation of the CHARMM-deMon interface, the electrostatic interaction of the QM/MM coupling is calculated by deMon2k, whereas the van der Waals interaction is computed by CHARMM. The MM charges and van der Waals parameters are copied from the CHARMM27 force field. In a special version of deMon2k, the vdW interaction can also be calculated. This was implemented to perform the macro/microiteration optimization scheme [27,28]. It is available upon request.

In addition to the normal electrostatic interaction, a special treatment using the Drude polarizable force field is described below. Regarding the vdW parameters, Friesner and co-workers [29] have re-optimized the vdW parameters for amino acids in their QM/MM implementation. More recently, Mulholland and co-workers re-optimized vdW parameters from CHARMM 27 for nucleic acids with respect to the B3LYP DFT method [30]. Their results indicated that, for QM/MM investigations of nucleic acids, the standard force field vdW parameters might not be appropriate for atoms treated by QM. However, Cui and co-workers have tested three sets of vdW parameters and concluded that the QM/MM energetics were not sensitive to the vdW parameters and efforts to improve QM/MM accuracy should focus elsewhere [31].

In the boundary treatment, the link-atom scheme is employed. A hydrogen is placed between the QM and MM boundary atoms. The position of the link atom (LA) is defined as a function of the positions of the QM boundary atom (QBA) and the MM boundary atom (MBA) in Cartesian coordinates:

(14) $${\vec{R}}_{LA}={\vec{R}}_{QBA}+\alpha ({\vec{R}}_{MBA}-{\vec{R}}_{QBA})$$

as is evident from:

(15) $$\alpha =\frac{{\vec{R}}_{QBA}-{\vec{R}}_{LA}}{{\vec{R}}_{MBA}-{\vec{R}}_{QBA}}$$

where α can be held constant as the ratio of the equilibrium bond lengths of QBA-LA and MBA-LA [32].

Further technical details on the interface development testing and implementation have been provided previously [6,33] and we will focus below on the conceptual implementation and examples of QM/MM interface applications.

### 3.1. Tested Force-Fields and General Details of MD Simulations with CHARMM-deMon2k

The QM-MM interface is fully functional with standard CHARMM-37 force-field releases for simulations with non-polarizable potential functions. We also tested and confirmed compatibility with the developed polarizable Drude force-field. It is worthwhile to describe an approach for including polarization response within the Drude model. It builds on introducing an inducible dipole by adding a fictitious Drude particle with opposite sign attached to a heavy atom by a harmonic spring [34]. The resulting electrostatic potential for a system containing Drude particles can be expressed as:

(16) Eelec=∑i∑j<iqiqjrij+(∑i∑j'qiqj'rij'+∑i∑j'<i'qi'qj'ri'j')+12∑α'kα'dα'2

where a prime denotes Drude particles. The last term represents the oscillator self-energy expressed with a force constant k_α_ related to the site’s polarizability (α).

The electric field between classical and QM systems allows for adjustments in the positions of heavy atoms and fictitious Drude particles thus capturing the induced polarization in the MM and QM systems. A comparison of additive and polarizable force-fields was performed for water clusters by Lev *et al.* [33]. In all the simulations described below, simulations with non-polarizable force-fields were performed using the SHAKE algorithm for all bonds involving hydrogen atoms except QM atoms in the system. All MD QM/MM simulations were performed using a modified version of CHARMM c36a4 and c38b1 interfaced with deMon 4.2.1 (modified) [6,33]. The “gukini.src” source file, which contained the CHARMM/GAMESS and CHARMM/Q-Chem [25,35] interfaces, was modified to include deMon2k as the external quantum chemistry program. The positions of auxiliary Drude particles (attached only to heavy atoms) were propagated via an extended Lagrangian formalism through the assignment of a small mass (0.4 amu) at low temperature (1 K) using a separate thermostat. The VV2 integrator and the Langevin thermostat [34,36] were used for all simulations involving polarizable models and can be used for all simulations with classical force-fields.

### 3.2. Free Energy Perturbation with CHARMM-deMon2k: Application to Ion Solvation

One of the main goals of interfacing CHARMM and deMon2k was to enable free energy perturbation (FEP) simulations for particles of interest located in the QM region. To achieve this goal we have used the following protocol for slowly changing the system from one state to another through a number of intermediate alchemical steps. The Hamiltonian controlling this perturbation along the chosen path can be written as:

(17) $$H(\lambda ,t)=\lambda H_{A}(t)+(1-\lambda )H_{B}(t)$$

where subscripts A and B correspond, respectively, to the initial and final states of the system.

The corresponding Hamiltonians (H_A_ and H_B_) encompass all of the objects in the mixed system (QM/MM); the approach is also known as the dual-topology scheme [37]. The perturbation parameter (λ) is used to propagate the system of interest from one state to the other. The parameter lambda varies over a range of values between 0 and 1, with λ = 1 representing state A and λ = 0 representing state B of the perturbation path. The integration along the perturbation path allows for direct measurement of the relative free energy difference between state A and B in the system provided the sampling is complete.

To illustrate the performance of different basis-sets, we considered a water droplet large enough to cover at least two solvation layers around an ion (21 molecules). The number of QM water molecules in this droplet was varied from 6 to 16. The perturbation coordinate is the one that converts our system from a water cluster with one ion, say Na^+^, to a water cluster with another ion, K^+^, thus allowing the simultaneous mapping of structural re-organization of the solvent shell around an ion and the estimation of the relative free energy difference [38]. We found that Lennard-Jones parameters corresponding to the interaction energies between QM and MM particles have to be adjusted as described previously by Rowley and Roux [39]. Table 1 compares the performance of QM/MM FEP simulations for a system containing 16 QM waters and 234 MM waters for two perturbation pairs e.g., Na^+^/K^+^ and Cl^−^/Br^−^ in comparison to available experimental data. It is essential to start from a well-equilibrated state and therefore all of the systems for the QM/MM DFT calculations were first equilibrated with classical MD simulation (2 ns per window) and then re-equilibrated for 1 ps per window, prior to the production run of the QM/MM FEP λ-windows. Forward and backward simulations were performed for all of the systems studied with 11 λ-windows connecting the initial and final states of the system.

**Table 1:** FEP results for monovalent ion pair perturbation in comparison to experimental data. The free energy difference is presented in kcal/mol. Standard deviations for WHAM (Weighted Histogram Analysis Method) calculations were estimated using block averaging over three blocks.

System	Functional/Basis-Set	Sampling	∆∆G
**Cl-/Br- Perturbation**			
16 QM + 234 MM	PBE98-PBE/def2-TZVPPD	110 ps	6.5 ± 0.2
**Available Experimental Data**			
Extra Thermodynamic Hypothesis	Schmid *et al.* [40]		6.5
Aqueous Cluster Measurements	Tissandier *et al.* [41]		6.4
Aqueous Cluster Measurements	Klots [42]		3.3
Electrochemical Measurements	Gomer *et al.* [43]		5.3
**Na+/K+ Perturbation**			
16 QM + 234 MM	PBE98-PBE/ DZVP-GGA	~110 ps	−21.5 ± 0.2
**Available Experimental Data**			
Extra Thermodynamic Hypothesis	Schmid *et al.* [40]		−17.4
Aqueous Cluster Measurements	Tissandier *et al.* [41]		−17.2
Aqueous Cluster Measurements	Klotts [42]		−17.6
Electrochemical Measurements	Gomer *et al.* [43]		−17.6

The main conclusion drawn from the data in Table 1 and extensive comparisons of different basis sets [38] is that the overall correlation between QM/MM FEP simulations and available experimental measurements is very encouraging. The results for QM/MM FEP simulations with the CHARMM-deMon2k interface are also in excellent agreement with previously published CPMD [44] and hydration studies [45].

### 3.3. Hamiltonian Replica Exchange and the QM/MM Interface

Exhaustive sampling along a chosen reaction coordinate is a requirement for accurate estimation of free energies. Given the short time scales generally accessible to QM/MM this may be a very serious limitation. To circumvent the problem in free energy simulations, we rely on the method known as Hamiltonian Replica Exchange (H-REMD) that can be used along the FEP coordinate to enrich the sampling. In a free energy calculation with umbrella sampling or FEP or any other propagation method, considering *N* copies of a system that are identical except for some differences regarding a small number of reaction parameters, it is possible to make lists of these sub-states such that the difference in the parameters is smallest for the nearest neighbors in the list. The FEP/H-REMD [46] algorithm periodically exchanges coordinates between replicas. The probability of exchange is written as:

(18) $$P\left(q_{i}\to q_{j}\right)=\min\left\{1,e^{-\left[U_{i}\left(q_{j}\right)+U_{j}\left(q_{i}\right)-U_{i}\left(q_{i}\right)-U_{j}\left(q_{j}\right)\right]}\right\}$$

where U is the potential energy and subscripts *i* and *j* represent the replica number.

The rule for attempted exchanges during the replica-exchange MD simulation can be arbitrarily defined. Let’s illustrate it for the case of an even number of replicas. First, there is an attempt to exchange between the members of the list and their nearest neighbors according to the odd ↔ even rule, *i.e.*, 1 ↔ 2, 3 ↔ 4, . . . (*N* − 1) ↔ *N*; then, there is an attempt to exchange their nearest neighbors according to the even ↔ odd rule, *i.e.*, 2 ↔ 3, 4 ↔ 5, . . . (*N* − 2) ↔ (*N* − 1). Each neighboring exchange means that all the numerical values of the parameters of the members, or equivalently, instant configurations are simply swapped. In principle, a single list would allow swapping systems only with 2 nearest neighbors, but it will be too restrictive for multi-dimensional sampling space. The results collected in Table 1 were obtained with H-REMD simulations. We found that QM/MM FEP for a pair of anions combined with H-REMD led to significant improvement in computed relative free energies of hydration, while results for cation pairs remain virtually the same [38].

### 3.4. FIRES Separation: Flexible Boundaries between QM and MM Regions

One of the common challenges in treating solvation effects with QM/MM decomposition is diffusion of QM waters into the MM region. The use of simple harmonic constraints to keep water inside the QM region would lead to problems with solvent density at the interface and, overall, perturb the distribution of states accessible to the system. We built on the previously developed separation technique intended to reduce the dimensionality in classical simulations known as the Spherical Solvent Boundary Potential (SSBP) [47].

The Flexible Inner Region Ensemble Separator (FIRES) method was used to separate the system into QM and MM regions [39]. In any system with two explicitly defined regions made of solvent molecules, the configurational integral can be exactly separated into two parts with n molecules in the inner shell and N molecules in the outer shell. This separation is independent of the choice of potential function [48]. The boundaries between QM and MM regions of the system in FIRES are deformable and a restraining force is imposed to prevent MM molecules from penetrating the sphere defined by radius R_in_. The constraint also ensures that the QM solvent molecules remain nearest to the ion. A force constant of 50 to 500 kcal·Å^−2^ was found to be effective at maintaining this condition [39] in studies of ion solvation.

(19) $$R_{in}=MAX\left(\left|r_{1}\right|,\ldots ,\left|r_{N}\right|\right);\;E_{FIRES}=\overset{N}{\underset{j=n+1}{\sum }}\frac{k_{FIRES}}{2}{\left(r_{j}-R_{in}\right)}^{2}$$

**Figure 6:**
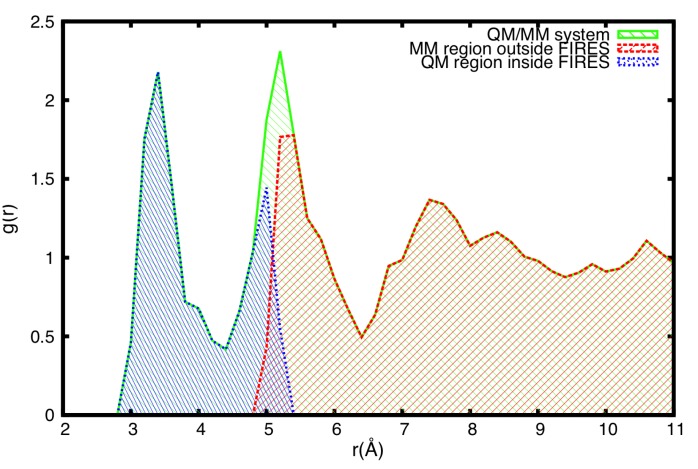
QM/MM Decomposition of the system with FIRES. The oxygen-ion Radial Distribution Function and its quantum and classical components for solvated Cl^−^. The droplet is composed of 16 QM and 250 MM waters. The flexible boundaries were implemented by the FIRES protocol.

To illustrate the usefulness of such a separation we will consider two examples. In one we will focus on the ion-water pair radial distribution function (RDF) shown in Figure 6.

The QM/MM FIRES protocol enables a proper position and coordination distribution in QM, QM/MM and MM regions of the system. However, it requires prior sampling of the system to determine the size of the solvation shell required to describe the process of interest. This can be done routinely by using classical simulations first.

### 3.5. Example of FIRES for QM/MM Simulations of Biological Molecules in Water

**Figure 7:**
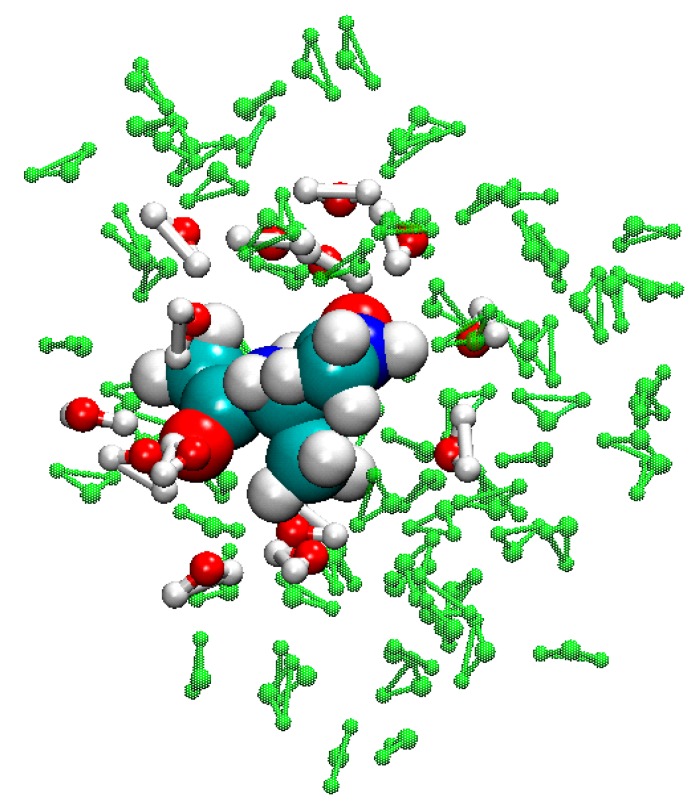
Alanine dipeptide solvated in water. The atoms comprising the QM region (alanine-dipeptide and first-contact shell water molecules within *R*_inner_ ≤ 3.0 Å) are shown as VDW spheres and red-and-white ball-and-stick models, respectively, while MM waters are shown as green ball-and-stick molecules.

Figure 7 shows an alanine dipeptide solvated in water [49]. There are 128 water molecules and the dipeptide, which are divided into MM and QM regions. A spherical harmonic constraint is applied to maintain all of the atoms within a sphere with respect to a fixed atom, whose water density is approximately equal to unity. In the QM region, there is a thin layer of 15 water molecules, which are found within 3.0 Å around the alanine dipeptide. This thin layer of water is kept near the dipeptide during simulations by means of the FIRES restraining protocol so the QM region can dynamically change its shape but remains well defined. FIRES keeps the number of water molecules in the thin layer unchanged during simulations by (i) defining the largest inner sphere, whose radius [*R*_inner_ = max(*r_m_*)] is the largest distance between the oxygen atoms (*r_m_*) in the thin layer and the middle nitrogen of the dipeptide, which is fixed during simulations to avoid any drifting without creating any artifacts; (ii) any outside water molecules with distances *r_n_* with respect to the nitrogen atom, which do not belong to the thin layer but enter the inner sphere, are pushed outside of the inner sphere by spherical harmonic potentials *k*_FIRES_(*r_n_* − *R*_inner_)^2^/2; (iii) the total force due to the harmonic potentials is also applied to the oxygen atom having the distance equal to *R*_inner_ with the opposite direction. Note that the water layer may not have a spherical shape. We find that *k*_FIRES_ of about 10 kcal/mol/Å^2^ can maintain the water molecules near the dipeptide and does not artificially push water molecules too close to the dipeptide or break valence bonds in the water molecules.

## 4. In-deMon2k QM/MM

Nowadays, most QM/MM calculations are performed either from a QM driver program (CPMD, Gaussian 09) or from an MM master code (CHARMM, Gromacs). Some interface codes (ChemShell, Cuby, PUPIL) [50,51,52,53] also exist, allowing connections between chosen QM and MM programs. If deMon2k is used as the driver for QM/MM calculations a unified input syntax is available along with a generalized output format. At this point it is also important to note that deMon2k QM, QM/MM and MM optimizations, molecular dynamics (MD) and frequency analyses use the same program branches and, therefore, are directly comparable. Moreover, electronic properties are readily available for QM and QM/MM calculations. In short, the in-deMon2k QM/MM implementation provides a user-friendly input for MPI QM/MM calculation with up to around 50,000 atoms.

Our target applications include the study of structures and properties of a reasonably large QM subsystem with up to around 500 QM atoms surrounded by some thousands of MM solvent molecules. Conformational free energies or reaction energies can be evaluated for systems of this level of complexity [54,55,56]. Dynamical properties of a QM system in an MM solvent are also of great interest when accompanied by the evaluation of spectroscopic properties along the dynamics [57]. Indeed, these two types of application are facilitated when the solvent (or any defined MM subsystem) is treated within the same computational framework (optimization, molecular dynamics *etc.*) as the QM sub-system. This is what has been achieved by incorporating MM force field routines into deMon2k. So far the OPLS-AA force field [58] has been implemented.

### 4.1. The in-deMon2k QM/MM Implementation

A single input file is used, taking care of the 3 types of calculations (QM, QM/MM, MM). It includes keywords and geometries. The QM, QM/MM and MM energies and derivatives are calculated separately but used together in geometry optimizations, molecular dynamics simulations and frequency analyses. As a result, free energy changes between conformers in a QM system embedded in MM solvent molecules can be obtained easily using the minimum free energy path method [59]. The QM and QM/MM frequencies are calculated by numerical differentiation of the corresponding analytic energy derivatives. MM frequencies are obtained from the analytical second derivatives.

The program can also be used for QM/MM calculations of a large MM molecule embedding a small QM site of interest with one or more bonds involving a QM-MM bond. In this case, a one-electron quantum capping potential can be used for the border QM atom, the MM border atom being an ordinary MM atom. For the capping potentials efficient half-numerical effective core potential (ECP) and model core potential (MCP) integral routines are available in deMon2k [60]. The capping potential is used in combination with an optimized basis set [61,62]. The advantage of this approach is that optimization or molecular dynamics can be performed per se without adding spurious atoms to the system.

The QM/MM energy in deMon2k is given by:

(20) $$E=E^{QM}+E^{QMMM}+E^{MM}$$

The QM energy, $\;E^{QM}$, consists of two parts, the ADFT SCF energy, $\;E^{SCF}$, as given by Equation (7) with the asymptotic expanded core Hamiltonian elements of Equation (5) and the MM augmented nuclear repulsion energy,

(21) $$E^{NN}=\;\overset{QM}{\underset{A>B}{\sum }}\frac{Z_{A}Z_{B}}{\left|\vec{A}-\vec{B}\right|}+\overset{QM}{\underset{A}{\sum }}\overset{MM}{\underset{D}{\sum }}\frac{Z_{A}Q_{D}}{\left|\vec{A}-\vec{D}\right|}$$

Because the so-defined QM energy contains all quantum mechanical terms plus the electrostatic embedding from the MM environment in $\;E^{SCF}$, the Kohn-Sham matrix elements can be defined as partial derivatives of this energy with respect to density matrix elements. For a more detailed description of the ADFT MinMax SCF we refer the interested reader to [10,14,18].

The second term in Equation (20) denotes the mechanical interaction energy between the QM and MM system. In the current developers’ version of deMon2k it is expressed as a Lennard-Jones potential of the form:

(22) $$E^{QMMM}=\;\overset{QM}{\underset{A}{\sum }}\overset{MM}{\underset{D}{\sum }}\varepsilon_{AD}\left[{\left(\frac{R_{AD}}{\left|\vec{A}-\vec{D}\right|}\right)}^{12}-2{\left(\frac{R_{AD}}{\left|\vec{A}-\vec{D}\right|}\right)}^{6}\right]$$

Here the $R_{AD}$ are combinations of the van der Waals radii of QM atom A and MM atom D. The parameter $\varepsilon_{AD}$ defines the depth of the Lennard-Jones potential and is taken by default from the MM force field. To do so the user needs to define the MM atom type for the QM atoms in the input.

The last term in Equation (20) denotes the MM energy that is given as:

(23) $$E^{MM}=E^{bond}+E^{bend}+E^{tors}+E^{urey}+E^{vdW}+E^{QQ}$$

The first four terms denote bond stretching, angle bending, dihedral torsion and Urey-Bradley energies. The calculation of these energy terms require molecular connectivity information that is either given by the user in the input or automatically generated on the basis of the distances between MM atoms. The last two terms in Equation (23) represent van der Waals and point charge interaction energies between the MM atoms. These terms are straightforwardly calculated over double index loops. Based on the algorithms described here the current MM implementation inside deMon2k can handle up to around 50,000 MM atoms.

### 4.2. Benchmarking the in-deMon2k QM/MM Implementation

**Figure 8:**
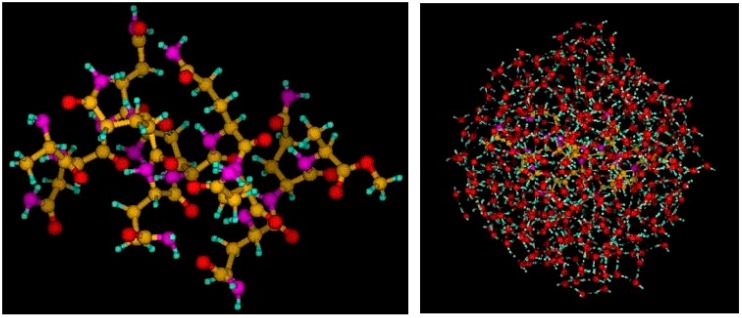
Structures of the A-Q9-A benchmark systems. On the left, the peptide with 179 QM atoms is depicted. On the right, the peptide dissolved in 440 MM waters is shown. Both structures are the starting points for the 1000 step MD runs discussed here.

In order to benchmark the in-deMon2k QM/MM implementation short MD runs of 1000 time steps of a free and solvated poly-glutamine sequence were performed. The QM calculated A-Q9-A peptide (A: Alanine; Q: Glutamine) structure was cut from the huntingtin-17Q X-ray structure, crystallized as a trimer (pdb file 3iow) [63]. The crystal coordinates display an α-helix structure. The N terminus was NH_2_ terminated whereas the C terminus was acylated, providing a neutral system with 179 QM atoms as shown in Figure 8, left. For solvation, this structure was then surrounded by 440 SPC MM water molecules as shown in Figure 8, right. Altogether, the system consists of 1499 atoms and can be treated fully QM with the DZVP basis used here and the A2 auxiliary function set [64]. For our benchmark calculations the local Dirac exchange [65] in combination with the VWN correlation [66] functional was used. The MD simulations were performed at 300 K in the canonical ensemble employing a Nosé- Hoover chain thermostat [67,68,69,70]. The time step was set to 1 fs.

Table 2 lists parallel CPU timings for these short MD runs with different numbers of processors (Intel^®^ Xeon^®^ X5550@2.67 GHz), from 16 to 96. The columns of this table refer to pure MM MD runs of the full system (MM), BOMD runs of the 179-atom QM system alone (QM peptide in the gas phase, Figure 8, left) employing the direct (QMd) and mixed (QMm) SCF algorithms and the corresponding hybrid QM/MM MD runs of the full system (QM peptide dissolved in 440 MM water molecules, Figure 8, right).

**Table 2:** Molecular dynamics timings [s] for benchmark calculations of the in-deMon2k QM/MM implementation. See text for details.

Number of Processors	MM	QMd	QMm	QM/MMd	QM/MMm
16	146	263,243	162,931	285,962	189,385
32	147	206,575	126,080	224,376	146,729
48	146	156,657	104,740	169,939	121,833
64	146	133,857	194,799	143,649	104,399
80	147	119,108	186,659	127,886	195,536
96	146	111,889	182,111	119,354	193,580

As can be seen from the first column of Table 2 the MM molecular dynamics of the full system takes less than 150 s, independent of the number of processors used. This is expected, due to the small system size. On the other hand, the gas phase BOMD timings of the peptide show a systematic decrease with respect to the number of processors. Note that these benchmark calculations were performed on a WestGrid cluster of Compute Canada and that load differences can alter the final timings by around 5%. Nevertheless, as expected from the discussion in Section 2.3, our benchmark results clearly show that BOMD simulations with the mixed SCF (QMm) are always faster than those with the direct SCF (QMd). This shows that the conclusions of Section 2.3 hold under real-life working conditions. As already discussed in the literature [15] a time plateau is reached with increasing number of processors due to the balance between communication and work load. As Table 2 shows the time difference between BOMD simulations with the direct and mixed SCF scheme is also decreasing with increasing number of processors. We attribute this to the very sophisticated work load balancing in the direct ERI calculation [15]. Nevertheless, even for the largest parallel BOMD with 96 processors, a speed-up of around 25% is still reached with the mixed SCF.

The hybrid QM/MM molecular dynamics (last two columns of Table 2) show on average an overhead of around 10,000 to 20,000 seconds with respect to the corresponding gas phase BOMD simulations. Note that this overhead usually reduces with increasing number of processors. As a result, the MM embedding increases total CPU times by 5% to 10%. This holds also for systems with much larger MM embedding (>100,000 MM atoms) [17] that are currently outside the system size range treatable with the in-deMon2k QM/MM implementation due to the asymptotic expansion of QM/MM embedding integrals discussed in Section 2.1. By and large the QM/MM hybrid molecular dynamics are only moderately more CPU time consuming than their pure QM BOMD counterparts but around 1000 times slower than pure MM molecular dynamics.

## 5. QM/MM with Cuby

### 5.1. Overview of Cuby

Cuby [51] is a software framework for computational chemistry written in the ruby programming language (the name stands for Chemistry in Ruby). Its main purpose is to simplify the development and implementation of complex computational protocols consisting of multiple calculations. The calculations themselves are carried out externally via interfaces to existing program packages. This allows one to connect calculations using different methods implemented in different codes. Cuby is developed as a general framework for automation of complex calculations and it is already used in several different projects. In this paper, we will describe the QM/MM functionality implemented in Cuby but it is not the only purpose of the code. While this makes the implementation of the QM/MM calculations less efficient, it makes it more flexible than dedicated QM/MM software. New computational protocols can be built out of existing modules without writing new code.

We begin with a brief description of the architecture of the framework. At the lowest level, there are libraries allowing an object-oriented description of the data we work with, most importantly molecular geometries and the results of calculations applied on them. Above this layer, there is the core of a framework that, using the information from a structured input file, builds the complex calculation out of independent modules, handling the individual calculations and tasks, and manages the communication between the modules. All the method-specific functionality is implemented in these modules. There are two types of them.

The first type of Cuby module is the “interface” which runs a calculation, the input is the molecular geometry and calculation setup and the output is the corresponding energy, gradient and other properties (as requested). Most interfaces call an external program to perform the calculation, in that case, the interface automatically builds the input for that program, runs it and parses the output to get the results. Some interfaces do the calculation themselves, this is used for simple calculations such as adding empirical corrections to the underlying potential. Finally, there are interfaces implementing composite calculations, calling other interfaces to get partial results, which are then combined. This is the case of the QM/MM implementation in Cuby.

**Figure 9:**
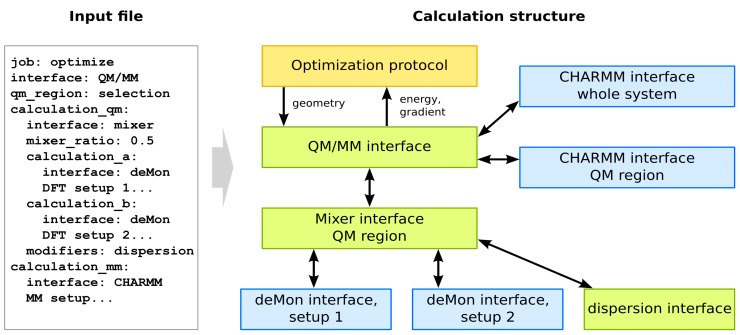
Structured input for Cuby (detailed setup for each module omitted for clarity) defines the structure of the Cuby calculation built from independent modules: the optimization driver (yellow), interfaces providing/combining calculations within Cuby (green) and interfaces to external software (blue).

The second type of Cuby module is an implementation of a computational “protocol”, a driver that operates on the potential calculated by the interface(s). Cuby implements all the commonly used protocols such as geometry optimization, harmonic vibrational analysis, molecular dynamics and more. The power of Cuby lies in the fact that all the modules can be arbitrarily combined, allowing combinations not available in the original software it calls. All these possibilities are available to the end user since the configuration of the modules and their connection is defined solely by means of a structured input file—no actual programming is needed. Figure 9 illustrates how such an input file for advanced QM/MM calculations (details are provided below) translates into a calculation built from the individual modules.

### 5.2. QM/MM Functionality in Cuby

Cuby uses a subtractive QM/MM formalism based on the ONIOM approach [32]. It allows one to use any external code without modifications because only regular calculations on complete molecules are performed. The QM/MM region is capped with hydrogen atoms when necessary. The QM/MM energy of the whole system is then defined as:

E _QM/MM_ (system) = E _MM_ (system) − E _MM_ (QM region) + E _QM_ (QM region) (24)

The gradient is calculated analogously, the force on the link atoms is projected to the real system according to ref. [32].

To achieve the electrostatic embedding, the QM region is calculated in a field of point charges automatically extracted from the MM calculation of the whole system (with options to exclude some charges and apply a distance cutoff to reduce their number). To avoid double counting of the electrostatics in the formula above, this has to be performed in both QM and MM calculations of the QM region. To obtain the complete QM/MM gradient, gradients on the point charges have to be included as well.

### 5.3. deMon2k and CHARMM Interfaces

The interface to deMon2k gives Cuby access to all the DFT functionality needed for QM/MM calculations, taking advantage of all the unique features of deMon2k discussed above. It also allows the insertion of custom options into the input it generates for deMon2k, making it possible to exploit any features of deMon2k. The interface supports deMon2k from version 2.5.4 but the more recent developer versions 4.2.X can be interfaced easily as all the necessary data are available in the QMMM file produced by deMon2k.

The CHARMM interface in Cuby performs not only the basic calculations of energy and gradient by calling CHARMM but it also handles the calculation of the interaction of the calculated system with the external point charges used in electrostatic embedding. Additionally, Cuby automates the setup of the CHARMM calculations. Provided an input geometry in CHARMM-compliant PDB format and the necessary library defining the forcefield for all the residues it contains, Cuby prepares all the parameter and input files it needs to perform the calculations.

### 5.4. DFT-D in Cuby

Noncovalent interactions often play an important role in complex molecular systems studied by QM/MM methods but the common DFT methods do not cover London dispersion. This deficiency is often corrected by an empirical correction potential which, despite its simplicity, allows one to achieve very good accuracy. Cuby contains its own implementation of the dispersion corrections for DFT, namely the DFT-D approach of Jurečka [71] and the more recent DFT-D3 approach of Grimme [72]. These corrections can be seamlessly added to the DFT calculation of the QM region in Cuby.

### 5.5. Automated QM/MM Setup in Cuby

A unique feature of Cuby is its optional automated setup of the QM region for a QM/MM calculation of a protein. Setting up a QM/MM calculation requires a lot of work in defining the QM region and building the parameters for the MM calculation of it. However, when the QM/MM boundary cuts only the protein backbone, the QM region can be built from complete residues and additional backbone caps that do not have to be reparameterized for a particular calculation. In such cases, Cuby can prepare the QM/MM calculation automatically starting only with a rough (e.g., distance-based) selection of the QM region. Then, all atoms needed to complete the residues and fill small gaps in the backbone are added and the termini are capped with the predefined cap residues. At present, the library of these caps is available only for the AMBER forcefield [73] but it can be easily adapted for CHARMM as well.

### 5.6. Other Cuby Functionality Used in QM/MM Calculations

The versatility of Cuby makes it possible to set up even more complex calculations. Various restraints can be added to the potential. The QM potential can be obtained by mixing two separate calculations (which can be called in parallel by Cuby), allowing for thermodynamic integration over the variable mixing ratio. A three-layer QM/MM can be set up by defining a QM/MM calculation where the QM calculation calls another instance of the QM/MM interface, allowing e.g., DFT/semiempirical/MM models.

### 5.7. Representative Applications

#### 5.7.1. Investigation of Copper Monooxygenases

**Figure 10:**
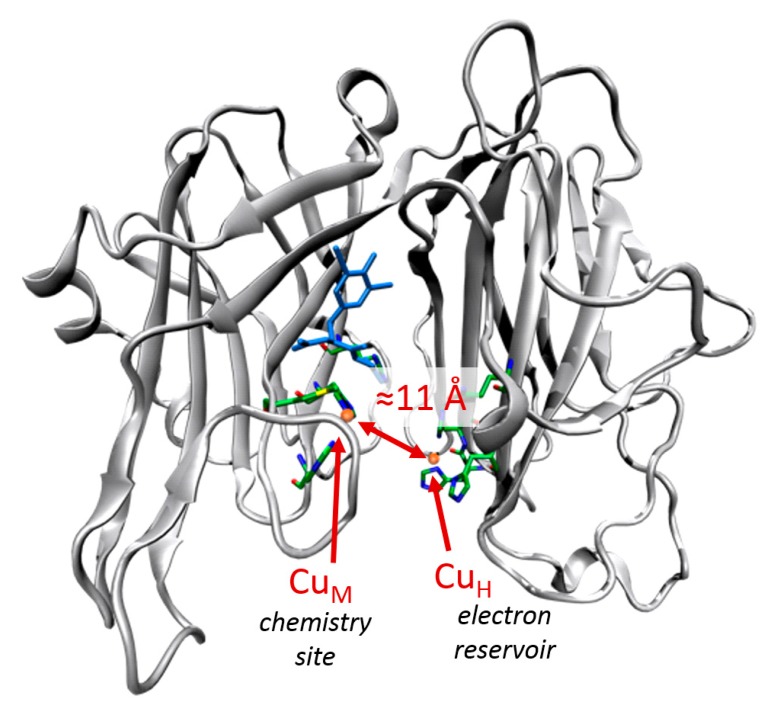
Crystal structure of PHM showing the two non-coupled copper sites separated by around 11 Å. The copper cations are shown in orange; the copper ligands in color with the licorice representation and the enzyme substrate in blue.

Metalloenzymes are natural biocatalysts capable of accelerating chemical reactions by several orders of magnitude compared to equivalent reactions in aqueous solution, often thanks to activating molecules such as N_2_, O_2_ or CO_2_. There is both fundamental and practical interest in unraveling the mechanisms of reactions catalyzed by metalloenzymes. Among them, the authors have been interested in the non-coupled copper monooxygenase Peptidylglyine a-Hydroxylating Monooxygenase (PHM) which is involved in the activation of neuropeptides [74]. PHM catalyzes the stereospecific hydroxylation of glycine-extended peptides (substrates) by activation of dioxygen molecules. PHM achieves catalysis using two remote copper active sites that are separated by a solvent cleft (Figure 10). At the beginning of the hydroxylation sequence both active sites (customarily denoted Cu_M_ and Cu_H_) are in the +1 oxidation state. It is known experimentally that each copper active site has to bring one electron to complete the 4-electron reduction of dioxygen (the remaining 2 electrons being formally provided by the substrate) [75]. Experimental evidence suggests that the “chemistry” takes place at the Cu_M_ active site while the Cu_H_ plays the role of an electron reservoir. Despite three decades of research the catalytic mechanism remains elusive and several mechanisms are still debated. In particular a prominent interrogation asks whether a one-electron reduced cupric-superoxide intermediate (Cu_M_(II)O_2_°^−^) is strong enough to break the substrate C-H bond; and, if so, what are the subsequent steps in the catalytic mechanism after the breaking of the C-H bond by the cupric-superoxide adduct?

**Figure 11:**
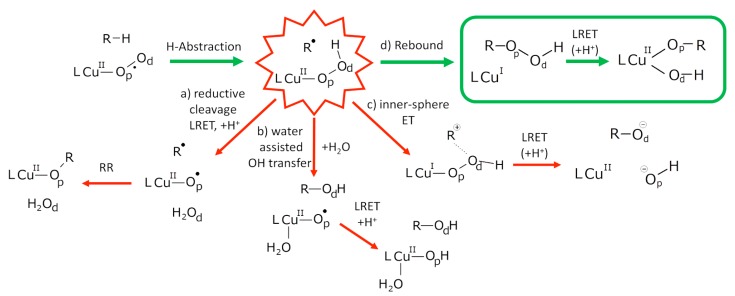
Evolution pathways for the cupric hydroperoxide intermediate: reductive cleavage a) [77], water assisted OH transfer b) [78], inner-sphere ET c) [79], or rebound mechanism d) [76]. R-H stands for the PHM substrate. L refers to the Cu_M_ ligands. LRET stands for Long Range Electron Transfer.

We recently conducted a computational investigation of the PHM mechanism by means of hybrid DFT/MM calculations coupled to Born-Oppenheimer MD simulations [76]. One of our main objectives was to determine the evolution of the cupric-hydroperoxide intermediate which is formed upon the H-abstraction of a hydrogen atom by the (Cu_M_(II)O_2_°^−^) adduct. Indeed various proposals have been advanced in the literature (Figure 11). For example, the cupric-hydroperoxide intermediate has been proposed to undergo a reductive cleavage upon injection of an electron from the Cu_H_ site, leading to two radicals that can recombine to form a hydroxyl function on the substrate (path a on Figure 11) [77]. Alternatively, a mechanism in which a hydroxyl radical leaving from the hydroperoxide ligand toward the substrate (assisted by coordination of water on the Cu_M_ site) has been proposed (path b) [78]. A third proposal suggests that an inner-sphere electron transfer from the Cu_M_ site produces a cationic substrate and a cuprous-hydroperoxide intermediate that is subsequently reduced upon ET from the Cu_M_ site (path c) [79]. Actually our BOMD simulations revealed yet another reaction pathway consisting of the radical recombination (rebound) of the hydroperoxide radical ligand with the radical substrate to form an alkylhydroperoxide intermediate (path d) [76]. This recombination takes place on the picosecond timescale. This timescale is imposed by the necessity for the hydroperoxide ligand to spin around the Cu-Op bonds so that the *proximal* oxygen atom (O_p_) faces the radical substrate (R°), hence enabling the formation of a covalent bond (ROOH). BOMD simulations allow one to understand how such fast reactive steps occur at the enzyme active site in contrast to current experimental approaches that cannot attain such degrees of time and space resolution. It can be argued that the rebound mechanism is likely to overtake in speed the alternative mechanisms. Therefore we could conclude on this study that, if the PHM mechanism in fact starts by an H-abstraction by a cupric-superoxide adduct, the resulting chemical intermediate is likely to be an alkylhydroperoxide. We further proposed that its reduction by the Cu_H_ site may lead to the hydroxylated substrate.

#### 5.7.2. Ultrafast Electron Transfer in Cryptochromes

Another illustration of the performance of the Cuby QM/MM interface is given in the context of the modeling of ultrafast electron transfer in a plant cryptochrome (AtCry1 from *Arabidopsis thaliana*). Cryptochromes belong to the family of flavoproteins which are involved in the regulation of circadian rhythms in plants and animals. They have also been proposed to play a role in magnetic field sensing by some species like drosophila or migratory birds [80]. Absorption of a blue photon by the flavin adenine dinucleotide (FAD) cofactor of cryptochrome initiates a long-range electron transfer from a tryptophan located on the surface of the protein (W324 in AtCry1 numbering) to the excited flavin (initially in its fully oxidized form FAD), which ultimately leads to the formation, after a series of proton transfers, of a long-lived radical pair Trp°/FADH°.

Time-resolved spectroscopy experiments [81] conducted on a related protein (*Chlamydomonas* photolyase homologue 1) suggest that electron transfer in AtCry1 takes place in less than a few hundreds of picoseconds (the kinetics may be even faster but no highly-resolved experiment is available). Such fast kinetics is best accounted for by the involvement of two tryptophan residues (W400 and W377) that mediate the long-range electron transfer between W324 and FAD*. This chain of three tryptophans is similar to that found in DNA-photolyases. Although mutation studies have shown the implication of the three tryptophans in the electron transfer [82], the details of the electron transfer mechanism are still unknown. Whether ET proceeds via superexchange or hopping is not established experimentally since no direct evidence of localization of the charge on the intermediate tryptophans could be reported so far with current experimental set-ups.

**Figure 12:**
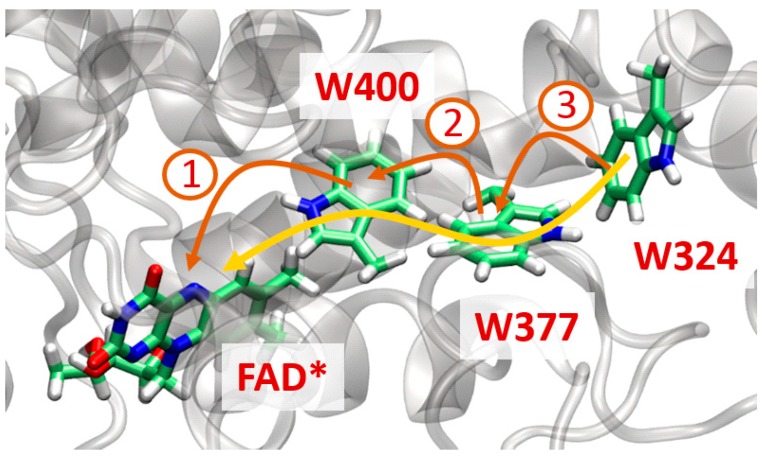
Photoactive site of *Arabidopsis thaliana* cryptochrome highlighting electron migration from W324 to the excited flavin cofactor (FAD*). Two limit mechanisms are shown: a fully coherent superexchange mechanism (in yellow) or an incoherent hopping mechanism (in brown).

Numerical simulations based on QM/MM schemes are valuable tools to decipher the physicochemical mechanisms governing such processes. When simulating electron transfers it is advantageous to adopt a diabatic description where each electronic state corresponds to the localization of the charge excess on a specific molecular fragment (here FAD, W400, W377 or W324). The constrained DFT methodology (cDFT) allows such definitions of non-adiabatic states at the DFT level. The implementation of cDFT in deMon2k and its compatibility with Cuby has been reported in a previous article [83]. In this review article we illustrate the use of Cuby to perform cDFT/MM MD simulations using deMon2k and CHARMM softwares to probe the transfer of one electron from W377 to W400°^+^. This electron transfer is the second step of the charge migration process in AtCry1 (see Figure 12), the first one being the transfer from W400 to FAD* (investigated recently in Ref [84]). The diabatic states (W377; W400°^+^) and (W377°^+^; W400) needed to describe the ET will be denoted CT1 and CT2 respectively. Two cDFT/MM MD simulations with different initial conditions have been carried out on the potential energy surface of state CT1. The QM partition encompasses the two tryptophan side chains and is treated with the PBE functional coupled to a DZVP basis set. MM charges located within 50 Å of the QM region have been included in the cDFT calculation. The initial configurations have been extracted from classical molecular dynamics simulations of AtCry1 performed in a previous study (see [84] for more details). At every time step (set to 1 fs) we estimate the vertical energy gap (defined as $\Delta E(t)=E_{CT2}-E_{CT1}$) and the electronic coupling ($H_{DA}(t)$) between CT1 and CT2. Both quantities are of primary interest in the determination of the ET kinetics.

**Figure 13:**
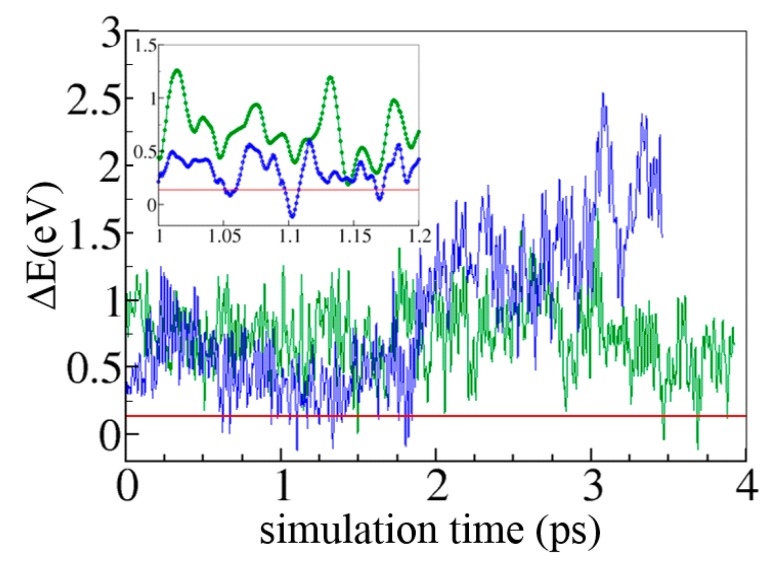
Evolution of the diabatic energy gap along two cDFT/MM MD trajectories carried out on the PES of state CT1. The horizontal red line indicates the value of the average electronic coupling obtained along the two simulations.

Figure 13 shows the evolution of ΔE along the two MD trajectories. In each case, ΔE fluctuates above zero. This means that state CT1 is more stable than state CT2 (which is consistent with the fact that the MD simulations are carried out on the PES of the CT1 diabatic state and that the environment thus adapts to the charge distribution of CT1). It is worth noting that significant fluctuations of (larger than 0.2 eV) take place on the femtosecond timescale (see inset graph). These fluctuations result from the internal dynamics of W377 and W400 and also from the variation of the positions of the environment charges. Despite the average lower energy of CT1, inversion of the relative stability of CT1 and CT2 happens frequently on the timescale covered by the MD simulations which can lead to population exchanges. The probability of exchange depends on the value of the electronic coupling which amounts to 0.16 eV on the average. Note that $H_{DA}$ is probably overestimated with the PBE functional used in these calculations [85], and should be either scaled down by an *ad hoc* correcting function or recomputed with hybrid functionals which are known to decrease cDFT electronic coupling values [86].

Longer MD simulations will be required to draw statistically meaningful results regarding the ET mechanism and these results will be reported in due course. However, these preliminary results demonstrate the practical feasibility of cDFT/MM simulations for modeling ultrafast ET of biological interest. All the quantities required to assess the applicability of common ET kinetic theories (Fermi Golden rule, Frank-Condon approximation, decoherence times...) are accessible with the method presented here. The graph in Figure 13 also suggests that a profitable coupling between cDFT/MM and non-Born-Oppenheimer MD algorithms like the surface hopping, Ehrenfest [87] or mixed quantum-classical Liouville approaches, is at one’s fingertips.

## 6. Concluding Remarks

We hope that this mini-review has shown some of the possibilities of using deMon2k for QM/MM calculations and simulations for interesting and complex systems. Each of the three implementations (CHARMM-deMon2k, in-deMon2k, and Cuby) has its pros and cons and is advocated by various workers, depending on the systems in which they are interested. CHARMM-deMon2k brings the power of CHARMM and its developer community to play. In-deMon2k is most powerful for larger QM regions and large but not huge MM regions. CUBY has the flexibility to bring in new MM (and other QM) techniques at will. We also hope that interested readers will visit the deMon2k web site [7] or contact one of the authors by email if they are interested in using the latest developers’ version.
